# Supplementary material for: Human connectome topology directs cortical traveling waves and shapes frequency gradients
Source: Nat Commun. 2024 Apr 26;15:3570. doi: 10.1038/s41467-024-47860-x (PMC11053146; doi:10.1038/s41467-024-47860-x)
Supplement: Supplementary file 1 — Supplementary Information [file 41467_2024_47860_MOESM1_ESM.pdf]

Supplementary Information

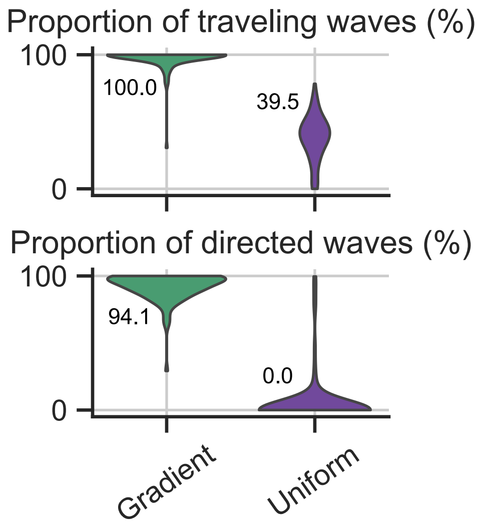

Supplementary Figure 1. Proportions of traveling waves and instrength-directed traveling waves in the 2D network models. The top row shows the proportion of traveling waves emerging in the 2D network models with gradient (green) and uniform (purple) instrength distributions. Violinplots show the distribution of proportions of traveling waves across 100 randomly initialized simulations of 10 s duration for each model (1 s transients removed). The median proportion is indicated next to each violinplot. Source data are provided as a Source Data file.

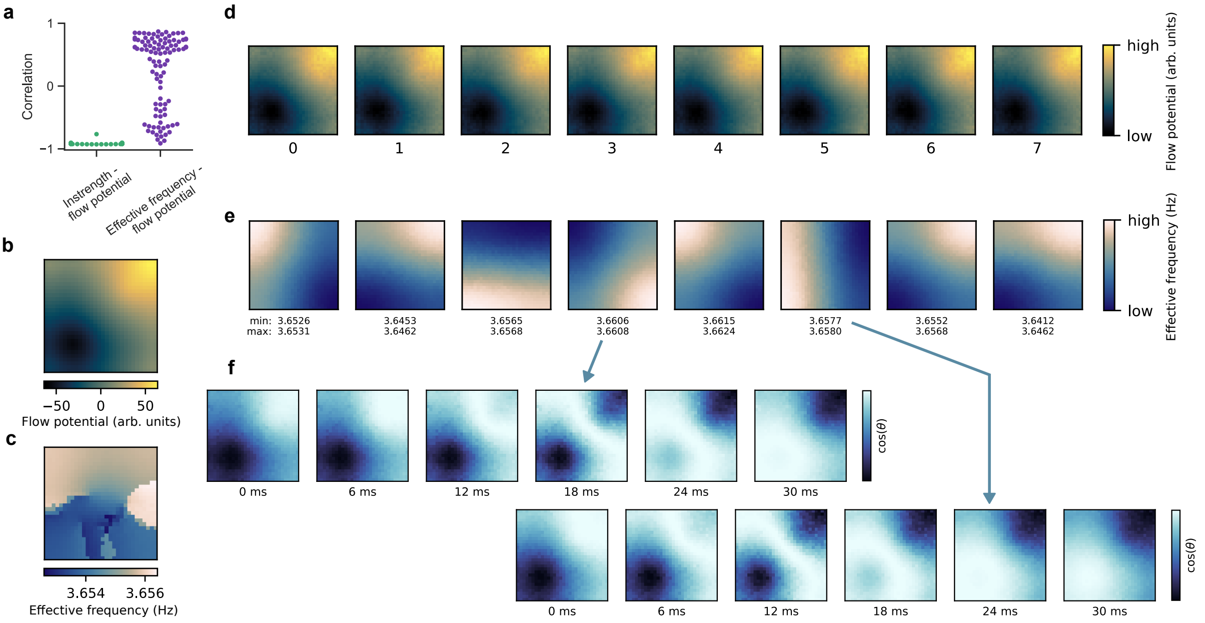

Supplementary Figure 2. 2D network model with inconsistent effective frequency maps. a Swarmplots of Instrength - flow potential and effective frequency – flow potential correlations across 100 randomly initialized simulations of 10 s duration (1 s transients removed). **b** The average flow potential indicates that traveling waves propagated along the instrength gradient. **c** The average effective frequency shows clusters of varying effective frequency due to distinct effective frequency patterns in individual simulations (see **e**). **d** Example wave potentials of eight individual simulations; all of them are consistent with instrength-directed traveling waves. **e** Example effective frequency patterns of the same eight simulations shown in **d**. Effective frequency patterns vary between simulations inconsistent with the wave potential. **f** Example timeseries of simulated traveling waves propagating along the instrength-gradient despite an orthogonal effective frequency pattern (top row) or opposing effective frequency gradient (bottom row). Source data are provided as a Source Data file.

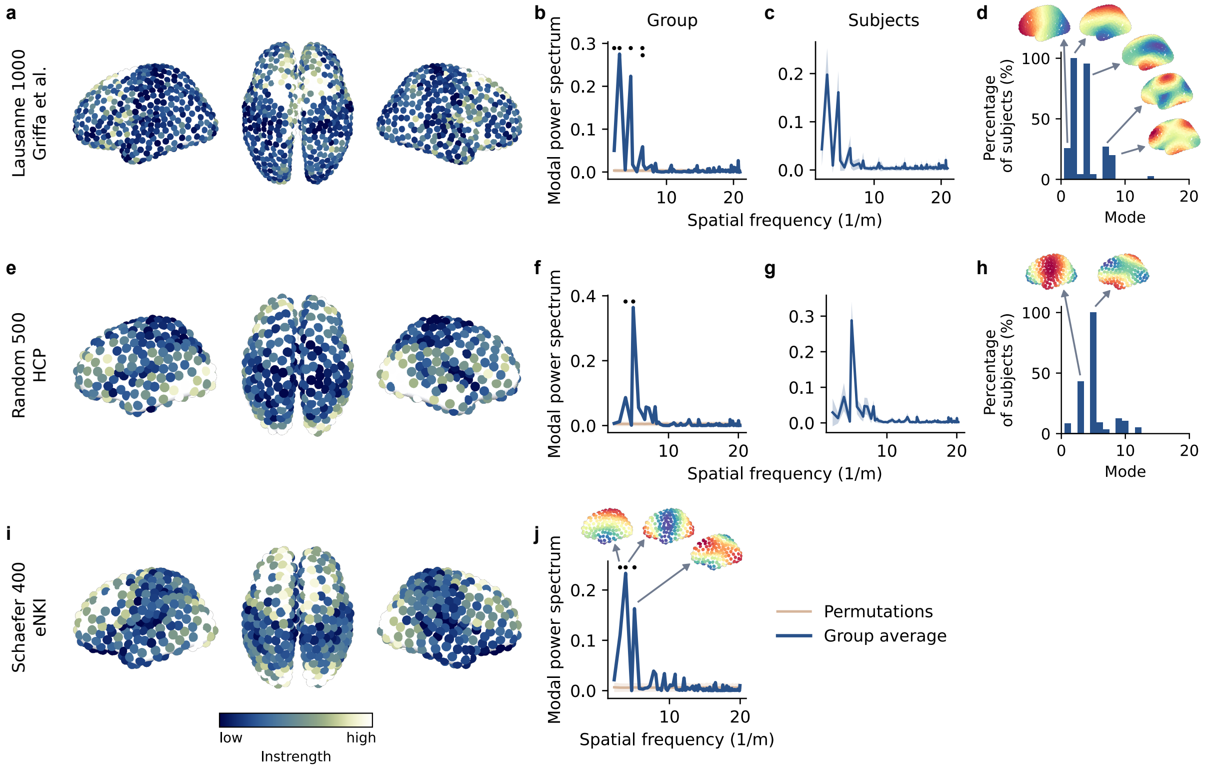

Supplementary Figure 3. Instrength gradients in the human connectome are a robust finding across distinct parcellations and cohorts. a Average instrength distribution across 70 young and healthy subjects (age: 28.8 +- 9.1 years, 27 females) estimated with deterministic tractography (https://zenodo.org/record/2872624#.Y-JarOzMKDU) for 1000 regions of the Lausanne atlas.[1] Instrengths were thresholded at the 5th and 95th percentiles. **b** Modal power spectrum of the group-averaged Lausanne1000/Griffa instrength pattern (blue). Statistical significance was assessed with one-sided permutation tests (n = 10,000 permutations; Bonferroni-corrected; baige line represents mean modal power of all permutations and shaded area the respective standard deviation; see Methods). **c** Average modal power of subject-level power spectra (thick blue line is the mean and the shaded area represent the standard deviation). **d** Percentage of subjects for which significant modes were identified. The five modes that were significant in the group-averaged connectome are shown. **e** Average instrength distribution derived from 972 participants of the HCP S1200 release (age: 28.7 ± 3.7, 522 females) for 500 equally sized regions of a random parcellation.[2] This data set is freely available from Zenodo (https://zenodo.org/record/4733297#.Y6w_LOzMJb8). Instrengths were thresholded at the 5th and 95th percentiles. **f** Modal power spectrum of the group-averaged Random500/HCP instrength pattern (blue). **g** Average modal power of subject-level power spectra (thick blue line is the mean and the shaded area represent the standard deviation). **h** Percentage of subjects for which significant modes were identified. The two modes that were significant in the group-averaged connectome are shown. **i** Average instrength distribution estimated from 369 subjects (age: 42.7+-17.9 years, 243 females) that participated in the Enhanced Nathan Klein Institute Rockland Sample[3] for the Schaefer parcellation with 400 regions. Processing details and the dataset are publicly available from EBRAINs (https://search.kg.ebrains.eu/instances/3f179784-194d-4795-9d8d-301b524ca00a). Instrengths were thresholded at the 5th and 95th percentiles. **j** Modal power spectrum of the group-averaged Schaefer400/eNKI instrength pattern (blue). The three modes that were significant in the group-averaged connectome are shown. Source data are provided as a Source Data file.

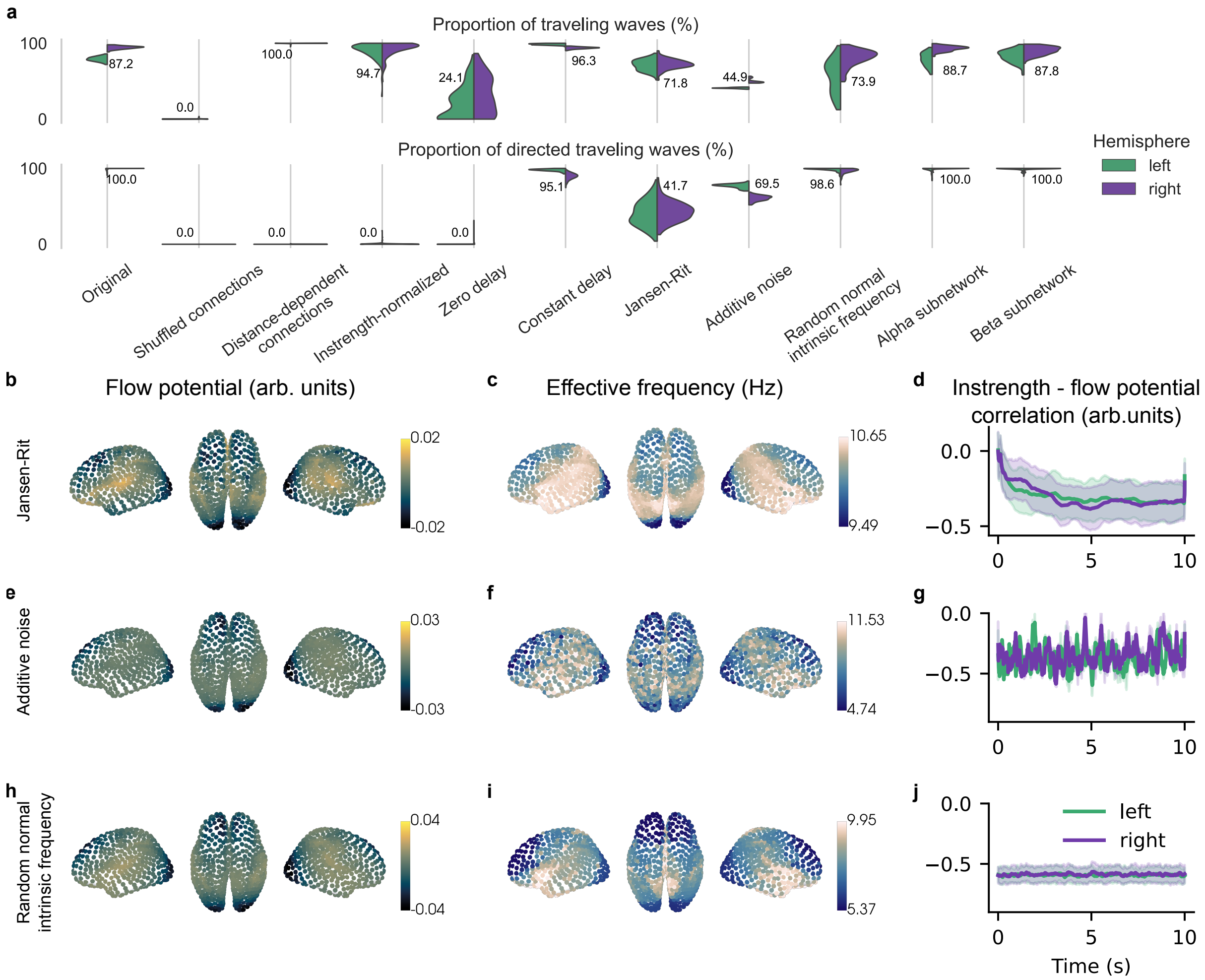

Supplementary Figure 4. Traveling wave proportions and additional control models. a Proportion of traveling waves emerging on all analyzed cortical network models and the proportion of waves directed by the original instrength gradient (across 100 randomly initialized simulations of 10 s duration with 1 s transients removed). Violinplots split by cortical hemispheres (left: green, right: purple) show the distribution of proportions. The median proportions are indicated next to each violinplot. **b** Average flow potential in Jansen-Rit model suggests that traveling waves propagate from temporal and parietal areas to frontal and occipital areas (100 simulations of 10 s duration with 1 s transients removed at the beginning and end). **c** Average effective frequency has a similar spatial pattern to the instrength distribution with faster oscillations around temporal and parietal areas and slower oscillations in frontal and occipital regions. Therefore, the relationship between instrength distribution and frequency patterns found with the Kuramoto model holds for the biologically more realistic Jansen-Rit model (see Supplementary Movie 9). **d** The instantaneous instrength – flow potential correlation across simulations evolves from zero to a stable inverse relationship for both hemispheres. This time course suggests that initially random activity organizes into traveling waves which eventually follow the instrength distribution of the connectome. **e** We tested if the cortical network model is robust against random additive noise with a standard deviation of 0.01 rad and evolved this model with a Heun stochastic integrator (see Supplementary Movie 10). The average flow potential correlated significantly with the instrength gradient ($r = -0.6$, $p < 0.01$). **f** Effective frequency strongly correlated with the instrength gradient ($r = -0.74$, $p < 0.01$). **g** The instrength - flow potential correlation dynamics show variable but instrength-directed wave dynamics. **h** We further tested if the

cortical network model is robust against random normally distributed intrinsic frequencies with a mean of 10 Hz and a standard deviation of 1 Hz (see Supplementary Movie 11). The average flow potential correlated significantly with the instrength gradient (r = -0.79, p < 0.01). **i** Effective frequency strongly correlated with the instrength gradient (r = -0.89, p < 0.01). **j** The instrength - flow potential correlation remains reflects instrength-directed waves. Source data are provided as a Source Data file.

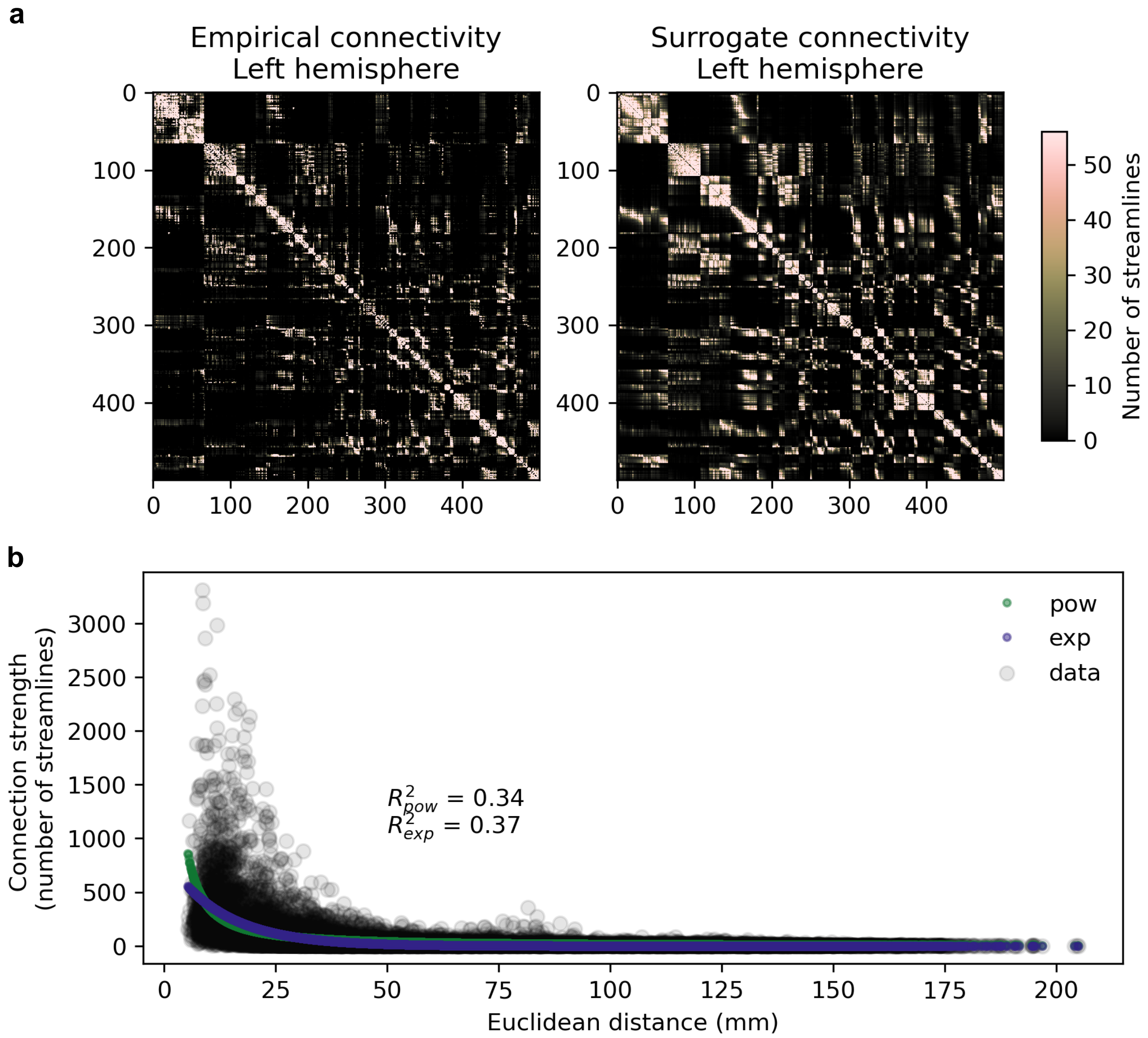

Supplementary Figure 5. Surrogate structural connectivity based on the empirical relationship between connection strength and euclidean distance. a Empirical average structural connectivity (left) within the left hemisphere (Schaefer 1000 region parcellation) estimated from 776 subjects that participated in the human connectome project (S900 release). Surrogate structural connectivity (right) synthesized using the relationship between connection strengths and euclidean distances between brain regions of the empirical structural connectivity. Both structural connectivity matrices were thresholded at the 95th percentile of the surrogate connectivity weights. **b** Relationship between connection strengths and euclidean distance estimated from the average structural connections between the 500 regions of the left hemisphere of the Schaefer atlas (gray circles). This relationship was fit with an exponential (purple) and a power-law (green) model. The exponential model resulted in a slightly better fit ($r_{exp}^2 = 0.37$ vs. $r_{power}^2 = 0.34$) and was thus used to synthesize the surrogate structural connectivity for our control model (see figure 5b - distance dependent connections). Source data are provided as a Source Data file.

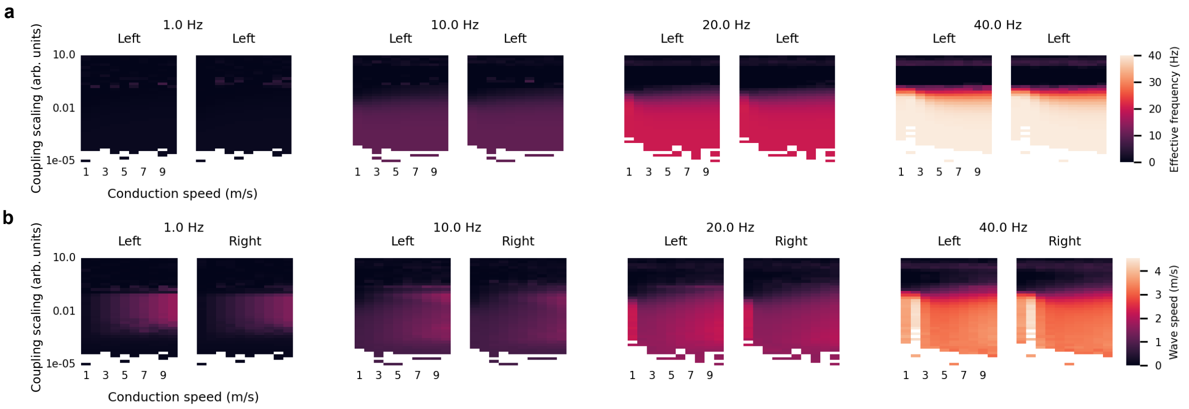

Supplementary Figure 6. Average effective frequency and wave speed in cortical network models. a Average effective frequency during traveling waves across cortical regions (10 simulations of 10 s duration for each parameter combination) was suppressed for higher coupling strengths as has been found in earlier studies.[4,5] White boxes represent parameter combinations at which no traveling waves were expressed across time and simulation. **b** Average traveling wave speed increased with intrinsic oscillation frequency (10 simulations of 10 s duration for each parameter combination) and was consistent with experimental observations.[6,7] White boxes represent parameter combinations at which no traveling waves were expressed across time and simulation. Source data are provided as a Source Data file.

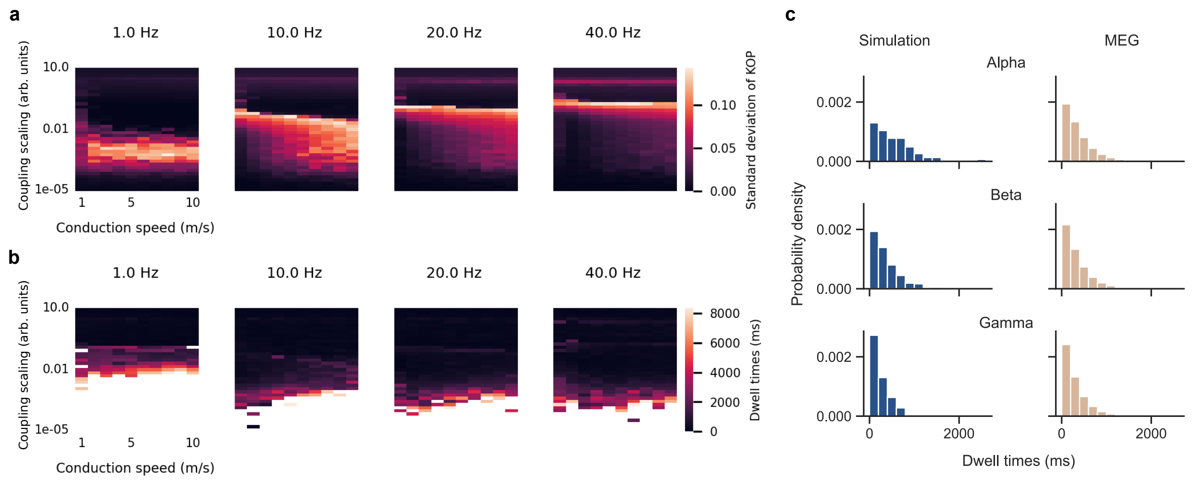

Supplementary Figure 7. Metastability in cortical network models with instrength gradients. a Standard deviation of kuramoto/synchronization order parameter (KOP) for various coupling strengths, conduction speeds in the delta, alpha, and beta bands. This measure has been frequently used to assess metastability in cortical dynamics.[8,9] **b** Dwell times of simulated cortical dynamics estimated by interhemispheric cross-correlation.[10] **c** Probability densities of simulated dwell times in cortical network models that fit the resting-state MEG functional connectivity estimated with the phase locking value best (right; see Figure 8) and densities of resting-state MEG for the alpha, beta, and gamma bands (n = 80 subjects). The cortical network models generated a right-skewed distribution with median dwell times of 430, 255, and 170 ms in the alpha, beta and gamma bands, while the median empirical dwell times were 270, 240, and 200 ms in the corresponding frequency bands. A Kolmogorov-Smirnov test indicated that the empirical and simulated alpha distributions were significantly different ($F = 0.209$, $p < 0.01$), while the beta ($F = 0.049$, $p = 0.671$), and gamma ($F = 0.077$, $p = 0.018$) distributions were indistinguishable. Source data are provided as a Source Data file.

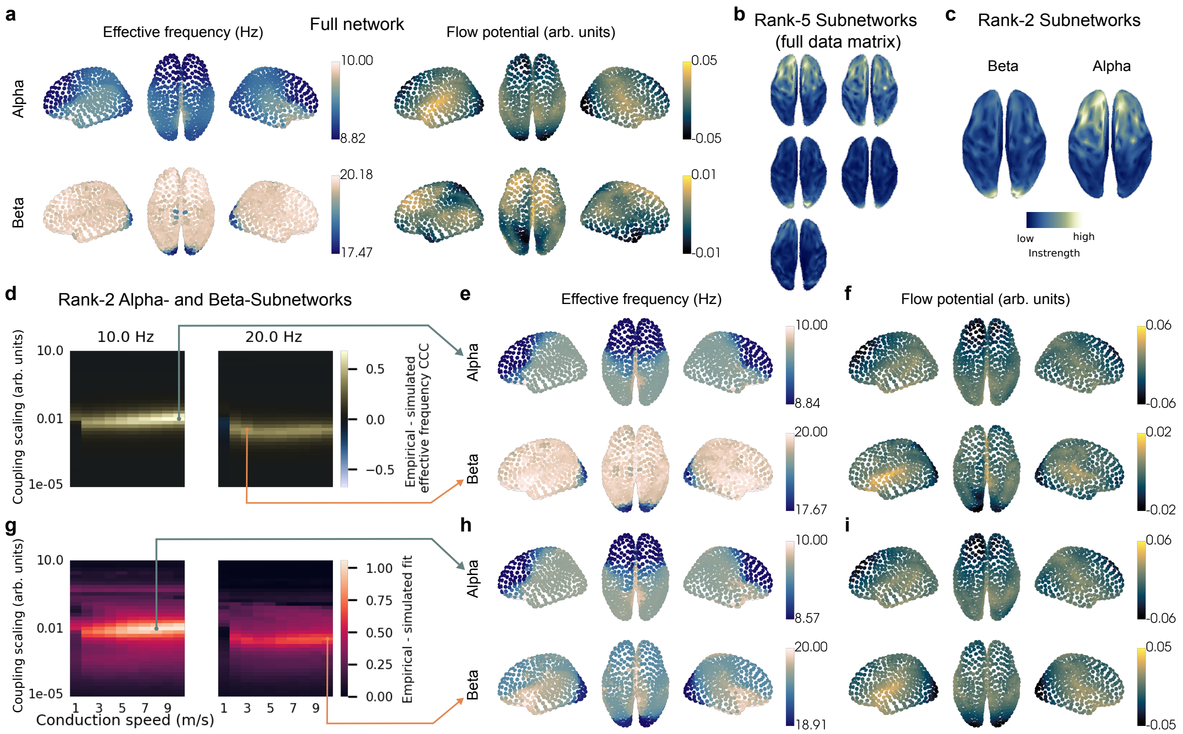

Supplementary Figure 8. Full network and subnetwork fit to resting-state MEG. a Average effective frequency and wave flow potentials in the alpha and beta bands for the full cortical network models at best EF CCC fit (averaged across ten randomly initialized simulations). **b** Average instrength of five subnetworks obtained through rank-5 nonnegative matrix factorization of the full data matrix (n = 776 subjects; best model out of 100 randomly seeded runs). **c** Beta- and alpha-subnetworks obtained through a rank-2 nonnegative matrix factorization (n = 776 subjects; best model out of 100 randomly seeded runs). **d** Parameter exploration of EF CCC fit for rank-2 alpha- and beta-subnetwork models (averaged across ten randomly initialized simulations). **e** Simulated alpha and beta effective frequency patterns at best EF CCC fit in **d** (averaged across ten randomly initialized simulations) **f** Wave flow potentials of alpha- and beta-subnetwork simulations at best EF CCC fit in **d** (averaged across ten randomly initialized simulations). **g** Parameter exploration of EF CCC + PLI-FC fit for rank-2 alpha- and beta-subnetwork models. **h** Simulated alpha and beta effective frequency patterns at best EF CCC + PLI-FC fit in **g** (averaged across ten randomly initialized simulations). **i** Wave flow potentials of alpha- and beta-subnetwork simulations at best EF CCC + PLI-FC fit in **g** (averaged across ten randomly initialized simulations). Source data are provided as a Source Data file.

Parameter	Description	Value	Units
A	Maximum amplitude of excitatory postsynaptic population response	3.25	mV
B	Maximum amplitude of inhibitory postsynaptic population response	22	mV
J	Average number of synapses between populations.	125	
a_1	Average probability of synaptic contacts in the feedback excitatory loop.	1	
a_2	Average probability of synaptic contacts in the slow feedback excitatory loop.	0.8	
a_3	Average probability of synaptic contacts in the feedback inhibitory loop.	0.25	
a_4	Average probability of synaptic contacts in the slow feedback inhibitory loop.	0.25	
a	Rate constant for postsynaptic population response to excitatory input	0.1	ms^{-1}
b	Rate constant for postsynaptic population response to inhibitory input	0.05	ms^{-1}
μ	Mean input firing rate to pyramidal population	0.22	ms^{-1}
ν_{max}	Half of the maximum population mean firing rate	2.5	ms^{-1}
r	Steepness of the sigmoidal transformation	0.56	mV^{-1}
v_0	Firing threshold (postsynaptic potential) for which a 50% firing rate is achieved.	5.52	mV
K	Global coupling scaling factor for network interactions	0.0025	

Supplementary Table 1. Parameters for Jansen-Rit cortical network model simulated with The Virtual Brain.

Supplementary References

1. Hagmann, P. *et al.* Mapping the structural core of human cerebral cortex. *PLOS Biol.* **6**, 1479–1493 (2008).
2. Arnatkeviciute, A. *et al.* Genetic influences on hub connectivity of the human connectome. *Nat. Commun.* **12**, 4237 (2021).
3. Nooner, K. *et al.* The NKI-Rockland Sample: A model for accelerating the pace of discovery science in psychiatry. *Front. Neurosci.* **6**, 152 (2012).
4. Niebur, E., Schuster, H. G. & Kammen, D. M. Collective frequencies and metastability in networks of limit-cycle oscillators with time delay. *Phys. Rev. Lett.* **67**, 2753–2756 (1991).
5. Nordenfelt, A., Wagemakers, A. & Sanjuán, M. A. F. Frequency dispersion in the time-delayed Kuramoto model. *Phys. Rev. E* **89**, 032905 (2014).
6. Muller, L. *et al.* Rotating waves during human sleep spindles organize global patterns of activity that repeat precisely through the night. *Elife* **5**, e17267 (2016).
7. Zhang, H., Watrous, A. J., Patel, A. & Jacobs, J. Theta and alpha oscillations are traveling waves in the human neocortex. *Neuron* **98**, 1269–1281 (2018).
8. Cabral, J. *et al.* Exploring mechanisms of spontaneous functional connectivity in MEG : How delayed network interactions lead to structured amplitude envelopes of band-pass filtered oscillations. *NeuroImage* **90**, 423–435 (2014).
9. Deco, G., Kringelbach, M. L., Jirsa, V. K. & Ritter, P. The dynamics of resting fluctuations in the brain: Metastability and its dynamical cortical core. *Sci. Rep.* **7**, 1–14 (2017).
10. Roberts, J. A. *et al.* Metastable brain waves. *Nat. Commun.* **10**, 1–17 (2019).
