# Supplementary material for: Human connectome topology directs cortical traveling waves and shapes frequency gradients
Source: Nat Commun. 2024 Apr 26;15:3570. doi: 10.1038/s41467-024-47860-x (PMC11053146; doi:10.1038/s41467-024-47860-x)
Supplement: Supplementary file 3 — Description of Additional Supplementary Files [file 41467_2024_47860_MOESM3_ESM.pdf]

Description of Additional Supplementary Files:

Supplementary Movie 1: Example traveling waves emerging in 2D network model with instrength gradient. The activity shown here is the $cos(\theta)$ of the instantaneous phase θ(t).

Supplementary Movie 2: Example traveling waves emerging in 2D control network model with uniform instrength. The activity shown here is the $cos(\theta)$ of the instantaneous phase θ(t).

Supplementary Movie 3: Example of traveling waves emerging in the cortical network model. The activity shown here is the $cos(\theta)$ of the instantaneous phase θ(t).

Supplementary Movie 4: Example activity emerging in control model with shuffled connection strengths. The activity shown here is the $cos(\theta)$ of the instantaneous phase θ(t).

Supplementary Movie 5: Example of traveling waves emerging in control model that preserves the relationship between connection strength and euclidean distance. The activity shown here is the $cos(\theta)$ of the instantaneous phase θ(t).

Supplementary Movie 6: Example activity emerging in control model with zero-delays. The activity shown here is the $cos(\theta)$ of the instantaneous phase θ(t).

Supplementary Movie 7: Example of traveling waves emerging in control model with constant delay. The activity shown here is the $cos(\theta)$ of the instantaneous phase θ(t).

Supplementary Movie 8: Example of traveling waves emerging in control model with instrength-normalized structural connectivity. The activity shown here is the $cos(\theta)$ of the instantaneous phase θ(t).

Supplementary Movie 9: Example of traveling waves emerging in control model with JansenRit neural masses. The activity shown here is the $cos(\theta)$ of the instantaneous phase θ(t).

Supplementary Movie 10: Example of traveling waves emerging in control model with additive gaussian noise. The activity shown here is the $cos(\theta)$ of the instantaneous phase θ(t).

Supplementary Movie 11: Example of traveling waves emerging in control model with random gaussian intrinsic frequency dispersion. The activity shown here is the $cos(\theta)$ of the instantaneous phase θ(t).

Supplementary Movie 12: Example of traveling waves emerging in the putative alpha band subnetwork. The activity shown here is the $cos(\theta)$ of the instantaneous phase θ(t).

Supplementary Movie 13: Example of traveling waves emerging in the putative beta band subnetwork. The activity shown here is the $cos(\theta)$ of the instantaneous phase θ(t).
