# Supplementary material for: Human connectome topology directs cortical traveling waves and shapes frequency gradients
Source: Nat Commun. 2024 Apr 26;15:3570. doi: 10.1038/s41467-024-47860-x (PMC11053146; doi:10.1038/s41467-024-47860-x)
Supplement: Supplementary file 17 — Reporting Summary [file 41467_2024_47860_MOESM17_ESM.pdf]

nature portfolio

Corresponding author(s): Dominik Koller
Petra Ritter

Last updated by author(s): Feb 29, 2024

Reporting Summary

Nature Portfolio wishes to improve the reproducibility of the work that we publish. This form provides structure for consistency and transparency in reporting. For further information on Nature Portfolio policies, see our Editorial Policies and the Editorial Policy Checklist.

Statistics

For all statistical analyses, confirm that the following items are present in the figure legend, table legend, main text, or Methods section.

n/a	Confirmed	
[ ]	[x]	The exact sample size (*n*) for each experimental group/condition, given as a discrete number and unit of measurement
[x]	[ ]	A statement on whether measurements were taken from distinct samples or whether the same sample was measured repeatedly
[ ]	[x]	The statistical test(s) used AND whether they are one- or two-sided<br>*Only common tests should be described solely by name; describe more complex techniques in the Methods section.*
[x]	[ ]	A description of all covariates tested
[ ]	[x]	A description of any assumptions or corrections, such as tests of normality and adjustment for multiple comparisons
[ ]	[x]	A full description of the statistical parameters including central tendency (e.g. means) or other basic estimates (e.g. regression coefficient) AND variation (e.g. standard deviation) or associated estimates of uncertainty (e.g. confidence intervals)
[ ]	[x]	For null hypothesis testing, the test statistic (e.g. *F*, *t*, *r*) with confidence intervals, effect sizes, degrees of freedom and *P* value noted<br>*Give P values as exact values whenever suitable.*
[x]	[ ]	For Bayesian analysis, information on the choice of priors and Markov chain Monte Carlo settings
[x]	[ ]	For hierarchical and complex designs, identification of the appropriate level for tests and full reporting of outcomes
[ ]	[x]	Estimates of effect sizes (e.g. Cohen's *d*, Pearson's *r*), indicating how they were calculated

Our web collection on statistics for biologists contains articles on many of the points above.

Software and code

Policy information about availability of computer code

Data collection	No software was used for data collection.
Data analysis	Open source: Python Python 3.8.12; The Virtual Brain 2.3; scipy 1.9.0; numpy 1.23.1; matplotlib 3.7.2; pyvista 0.41.1; LibIGL-Python-bindings 2.2.1; MNE-Python 1.0.3; MNE-HCP 0.1.dev12; BrainSpace 0.1.4; FreeSurfer 7.1.1 and 7.1.0; MRtrix 3.0.2 and 3.0; FSL 6.0; scikit-sparse 0.4.6; RcppML 0.5.5; R 4.3.1; reticulate 1.34.0<br>Custom software: Custom code for processing and analyzing data was written in Python 3.8.12 and R 4.3.1 using the above open source software packages and is freely available from osf.io/daq54

For manuscripts utilizing custom algorithms or software that are central to the research but not yet described in published literature, software must be made available to editors and reviewers. We strongly encourage code deposition in a community repository (e.g. GitHub). See the Nature Portfolio guidelines for submitting code & software for further information.

Data

Policy information about availability of data

All manuscripts must include a data availability statement. This statement should provide the following information, where applicable:

- Accession codes, unique identifiers, or web links for publicly available datasets
- A description of any restrictions on data availability
- For clinical datasets or third party data, please ensure that the statement adheres to our policy

Human Connectome Project S900 release data (structural and MEG resting-state) are publicly available from https://db.humanconnectome.org
The Schaefer atlas parcellation with 1000 regions is publicly available at https://github.com/ThomasYeoLab/CBIG/tree/master/stable_projects/brain_parcellation/Schaefer2018_LocalGlobal
The structural connectivity based on the Lausanne atlas is publicly available at https://zenodo.org/record/2872624
The structural connectivity based on the 400 region Schaefer atlas is publicly available at https://search.kg.ebrains.eu/instances/3f179784-194d-4795-9d8d-301b524ca00a
The structural connectivity based on a random parcellation with 500 regions is publicly available at https://zenodo.org/record/4733297

Research involving human participants, their data, or biological material

Policy information about studies with human participants or human data. See also policy information about sex, gender (identity/presentation), and sexual orientation and race, ethnicity and racism.

Reporting on sex and gender	We used open datasets and specify gender as reported by the original studies or open dataset descriptions (see Experimental Data). No sex- or gender-based analyses were conducted since the mechanisms described are expected to be generic.
Reporting on race, ethnicity, or other socially relevant groupings	We do not report these categories since they were not important to our analyses.
Population characteristics	The 776 Human Connectome Project subjects analyzed in our study had the following population characteristics (number of subjects / age range): 160 / 22-25, 339 / 26-30, 271 / 31-35, 6 / 36+; as per self-report 432 subjects are female and 344 subjects are male.
Recruitment	The Human Connectome Project recruitment process documents: "White non-Hispanic, Hispanic, Asian and African-American families will be invited to participate, to reflect the ethnic diversity of America" (https://www.humanconnectome.org/study/hcp-young-adult/project-protocol/recruitment)
Ethics oversight	Washington University in St. Louis Institutional Review Board (IRB); Medical Ethical Committee of the Charité Medical Center in Berlin.

Note that full information on the approval of the study protocol must also be provided in the manuscript.

Field-specific reporting

Please select the one below that is the best fit for your research. If you are not sure, read the appropriate sections before making your selection.

☒ Life sciences ☐ Behavioural & social sciences ☐ Ecological, evolutionary & environmental sciences

For a reference copy of the document with all sections, see nature.com/documents/nr-reporting-summary-flat.pdf

Life sciences study design

All studies must disclose on these points even when the disclosure is negative.

Sample size	We used publicly available datasets (see Experimental Data) for our analyses. Structural connectomes were estimated from 785 subjects that participated in the Human Connectome Project (S900 release) and had complete MRI data including structural MRI (T1w and T2w), diffusion-weighted MRI and all four sessions of resting-state fMRI. Nine subjects were excluded because of missing files that were necessary for our processing pipeline. We used the data of the remaining 776 healthy subjects to estimate structural connectomes. We used 89 subjects of the Human Connectome Project MEG cohort that had all necessary resting-state data for our analyses . Nine subjects were excluded from the final analyses due to fragmented recordings data. We used the remaining 80 subjects for our resting-state MEG analyses. Further details on sample size of the other publicly available datasets can be found in their associated publications (see Experimental Data).
Data exclusions	Nine subjects of the 785 subjects that participated in the Human Connectome Project S900 release could not be processed because files necessary for analysing the structural connectivity were missing. Nine subjects of the Human Connectome Project that participated in resting-state magnetoencephalography recordings were excluded because of fragmented (i.e. non-continuous) recordings.
Replication	We successfully replicated our finding of large-scale structural connectivity instrength gradients in two independent datasets (eNKI - Jung et al., 2022; Griffa et al., 2019) and one partially overlapping dataset (Human Connectome Project; Arnatkeviciute et al., 2021) that used a different processing pipeline and cortical parcellation to our study.

Randomization	Not relevant because we did not use experimental groups
Blinding	Not relevant because we did not use experimental groups

Reporting for specific materials, systems and methods

We require information from authors about some types of materials, experimental systems and methods used in many studies. Here, indicate whether each material, system or method listed is relevant to your study. If you are not sure if a list item applies to your research, read the appropriate section before selecting a response.

Materials & experimental systems

n/a	Involved in the study	
☒	☐	Antibodies
☒	☐	Eukaryotic cell lines
☒	☐	Palaeontology and archaeology
☒	☐	Animals and other organisms
☒	☐	Clinical data
☒	☐	Dual use research of concern
☒	☐	Plants

Methods

n/a	Involved in the study	
☒	☐	ChIP-seq
☒	☐	Flow cytometry
☐	☒	MRI-based neuroimaging

Plants

Seed stocks	*Report on the source of all seed stocks or other plant material used. If applicable, state the seed stock centre and catalogue number. If plant specimens were collected from the field, describe the collection location, date and sampling procedures.*
Novel plant genotypes	*Describe the methods by which all novel plant genotypes were produced. This includes those generated by transgenic approaches, gene editing, chemical/radiation-based mutagenesis and hybridization. For transgenic lines, describe the transformation method, the number of independent lines analyzed and the generation upon which experiments were performed. For gene-edited lines, describe the editor used, the endogenous sequence targeted for editing, the targeting guide RNA sequence (if applicable) and how the editor was applied.*
Authentication	*Describe any authentication procedures for each seed stock used or novel genotype generated. Describe any experiments used to assess the effect of a mutation and, where applicable, how potential secondary effects (e.g. second site T-DNA insertions, mosiacism, off-target gene editing) were examined.*

Magnetic resonance imaging

Experimental design

Design type	Structural and diffusion weighted MRI
Design specifications	Not relevant for our analyses
Behavioral performance measures	Not relevant for our analyses

Acquisition

Imaging type(s)	structural and diffusion MRI
Field strength	3T
Sequence & imaging parameters	diffusion: Spin-echo EPI, TR: 5520 ms, TE: 89.5 ms, flip angle: 78 deg, refocusing flip angle: 160 deg, FOV: 210x180 (RO x PE), matrix: 168x144 (RO x PE), slice thickness: 1.25 mm, 111 slices, 1.25 mm isotropic voxels, Multiband factor: 3, Echo spacing: 0.78 ms, BW: 1488 Hz/Px, b-values: 1000, 2000, and 3000 s/mm2<br><br>structural: T1w_MPR1, 3D MPRAGE, TR: 2400, TE: 2.14, TI: 1000, Flip angle: 8 deg, FOV (mm): 224x224, voxel size: 0.7 mm isotropic, BW: 210 Hz/Px
Area of acquisition	whole brain scans were used

Diffusion MRI ☒ Used ☐ Not used

Parameters: 3 shells (b=1000,2000,3000 s/mm2) with approx. 90 gradient directions per shell, no cardiac gating

Preprocessing

Preprocessing software	The preprocessed data as provided by Human Connectome Project was used, described in Glasser, M. F., Sotiropoulos, S. N., Wilson, J. A., Coalson, T. S., Fischl, B., Andersson, J. L., ... & Wu-Minn HCP Consortium. (2013). The minimal preprocessing pipelines for the Human Connectome Project. Neuroimage, 80, 105-124.
Normalization	Normalization followed the Human Connectome Project minimal preprocessing pipeline described in Glasser, M. F., Sotiropoulos, S. N., Wilson, J. A., Coalson, T. S., Fischl, B., Andersson, J. L., ... & Wu-Minn HCP Consortium. (2013). The minimal preprocessing pipelines for the Human Connectome Project. Neuroimage, 80, 105-124.
Normalization template	We resampled the Schaefer parcellation (2018; https://github.com/ThomasYeoLab/CBIG/tree/master/stable_projects/brain_parcellation/Schaefer2018_LocalGlobal) from the FreeSurfer average brain to the FreeSurfer subjects' individual space, followed by mapping this surface-based atlas to the volumetric image. We computed the structural connectivity from the tractography data and the parcellation image in subject space.
Noise and artifact removal	The preprocessed data as provided by Human Connectome Project was used as described in Glasser, M. F., Sotiropoulos, S. N., Wilson, J. A., Coalson, T. S., Fischl, B., Andersson, J. L., ... & Wu-Minn HCP Consortium. (2013). The minimal preprocessing pipelines for the Human Connectome Project. Neuroimage, 80, 105-124.<br>The Human Connectome Project's noise and artifact removal includes: readout, EPI, B0 inhomogeneity, eddy current and gradient distortion correction.
Volume censoring	No volume censoring was performed.

Statistical modeling & inference

Model type and settings	We used the structural and diffusion MRI data for tractography between Schaefer et al. (2018; see Data) atlas regions. The resulting structural connectivity (weights and fiber lengths) were analyzed and used in a cortical network model. Structural connectivity instrength was correlated with traveling wave flow potential and effective frequency resulting from the network models.
Effect(s) tested	We tested the strength of Spearman's correlation between structural connectivity instrength and traveling wave flow potential (scalar field that characterizes the direction of wave propagation) or effective frequency (oscillation frequency of neural mass models) generated by the cortical network model. We used permutation testing procedures to test those effects.

Specify type of analysis: ☒ Whole brain ☐ ROI-based ☐ Both

Statistic type for inference<br>(See Eklund et al. 2016)	Spatial correlations (instrength - wave potential or effective frequency) were tested between the 1000 regions of the Schaefer atlas (see Data) using permutation tests.
Correction	We corrected for spatial autocorrelation using spin permutation tests.

Models & analysis

n/a	Involved in the study
☐	☒ Functional and/or effective connectivity
☐	☒ Graph analysis
☒	☐ Multivariate modeling or predictive analysis

Functional and/or effective connectivity	We used the structural MRI images of the Human Connectome Project MEG cohort to conduct source reconstruction for MEG resting-state functional connectivity.
Graph analysis	We analysed the relationship (Spearman correlation) between instrength of the structural connectivity (sum of incoming connection strengths of the weighted graph) with traveling wave propagation (curl-free wave potential of the helmholtz hodge decomposition) and effective frequency gradients emerging in a cortical network model.<br>Furthermore, we decomposed the full structural connectivity graph into subnetworks using non-negative matrix factorization.
